# *Helicobacter pylori* modulates host cell survival regulation through the serine-threonine kinase, 3-phosphoinositide dependent kinase 1 (PDK-1)

**DOI:** 10.1186/s12866-015-0543-0

**Published:** 2015-10-21

**Authors:** Charles C. King, Marygorret Obonyo

**Affiliations:** Pediatric Diabetes Research Center, University of California, La Jolla, San Diego, CA 92093 USA; Department of Medicine, University of California, La Jolla, San Diego, CA 92093 USA

**Keywords:** *Helicobacter pylori*, PDK-1, Akt, AGS cells

## Abstract

**Background:**

*Helicobacter pylori* (*H. pylori*) infection affects cell survival signaling pathways including cell apoptosis and proliferation, which are considered risk factors for the development of gastric cancer when unregulated. In the present study, we investigated the effect of *H. pylori* infection on the phosphorylation state of 3-phosphoinositide-dependent kinase-1 (PDK-1), a master kinase that regulates phosphorylation of Akt (also known as protein kinase B, PKB) and cell survival.

**Methods:**

The activity of PDK-1 was examined in human gastric epithelial cells incubated in the presence or absence of different *H. pylori* strains. In addition, the role of *H. pylori* type IV secretion system and the mechanism of *H. pylori* effect on PDK-1 activity was examined.

**Results:**

In the presence of *H. pylori*, phosphorylation of the activation loop (serine 241) PDK-1 was rapidly lost suggesting that dephosphorylation of PDK-1 is a target for *H. pylori* to modulate cell survival. The extent of dephosphorylation was strain dependent with *H. pylori* 60190 being the most effective. *H. pylori* infection of gastric epithelial cells resulted in altered phosphorylation and degradation of Akt, suggesting that PDK-1 dephosphorylation affects cell survival pathways and thereby may contribute to disease pathogenesis.

**Conclusion:**

We propose that dephosphorylation of PDK-1 and the resulting changes to Akt phosphorylation is one of the mechanisms by which infection with *H. pylori* alter the balance between apoptosis and cell proliferation and identify a host molecular mechanism regulated by *H. pylori* that ultimately contributes to carcinogenesis. Our studies therefore provide insights into one of the mechanisms by which *H. pylori* infection contributes to gastric cancer by regulating the activity of a cell survival signaling pathway.

## Background

*Helicobacter pylori* infect over 50 % of the world’s population, causing inflammatory gastritis, peptic ulcer disease, and gastric cancer [1, 2]. The molecular mechanisms and signaling pathways underlying the transition from *H. pylori* infection to gastric cancer remain unclear. *H. pylori* virulence factors including a cytotoxin-associated gene A (*cagA*), pathogenicity island (PAI), and vacuolating cytotoxin A (VacA) [3–6] have been associated with severe *H. pylori*-related disease. Specifically, *H. pylori* strains harboring an intact cag PAI, encoding components of a type IV secretion system (T4SS), are associated with a high risk of gastric cancer [7, 8]. The T4SS, which is comprised of multiple transporters including the CagE protein, is used to inject the immunodominant CagA protein into the gastric epithelial cells. Therefore, CagA secretion depends on the expression of functional genes that encode the T4SS including *cagE*. Upon translocation into the eukaryotic host cell, CagA is tyrosine phosphorylated by Src family of kinases of the host, which lead to rearrangement of the host cell cytoskeleton termed the “hummingbird phenotype” and subsequent induction of cell scattering [9, 10]. This phenomena is widely considered to be important in neoplastic transformation [11, 12]. Phosphorylated CagA interacts with several major host cell signal-transduction pathways [13, 14] affecting cell morphology changes such as cell elongation and motility (see review [15]).

Several intracellular signaling pathways are activated upon *H. pylori* infection of host gastric epithelial cells [9, 10, 13–19]. Specifically, CagA interaction with SHP2 phosphatase and the Src family of kinases of the host cell are examples of how *H. pylori* is thought to hijack intracellular signaling pathways and potentially contribute to cancer development [20]. However, it is unlikely that signaling through these two pathways are exclusively associated with *H. pylori* pathogenesis. Therefore, we wanted to characterize gastric epithelial cellular signaling responses following *H. pylori* infection, with a focus on pro-survival signals from PDK-1, which has not been investigated in relation to *H. pylori* infection.

The best characterized cell survival signaling pathway is the PI 3-kinase/PDK-1/Akt pathway. Upon binding to activated tyrosine kinase receptors, phosphatidylinositol 3-OH-kinase (PI 3-K) phosphorylates inositol phospholipids at the D-3 position of the inositol ring to generate phosphatidylinositol 3,4-bisphosphate (PI-3,4-P2) and phosphatidylinositol 3,4,5-trisphosphate (PI-3,4,5-P3). These lipids serve as membrane docking sites for many pleckstrin homology (PH) domain-containing proteins, including PDK-1 and Akt. Phosphorylation of Akt by PDK-1 activates the enzyme which phosphorylates a number of pro-survival proteins [21, 22].

PDK-1 is a multi-domain enzyme that contains an amino terminal kinase domain and a carboxy terminal PH domain separated by a small linker region. The enzyme is constitutively autophosphorylated at position Ser 241 within the activation loop (kinase subdomain VIII) [23]. The primary function of PDK-1 appears to be that of a master regulatory protein kinase. PDK-1 phosphorylates the activation loop of AGC serine/threonine kinase family members including protein kinase A (cAMP-dependent protein kinase), protein kinase B (Akt), protein kinase C (PKC) isoforms, p70^S6^ kinase, and serum- and glucocorticoid-inducible kinase resulting in catalytic competence [24–31]. Phosphorylation of the activation loop in AGC protein kinases is thought to regulate access of substrates to the catalytic pocket. Phosphorylation of the specific activation loop, serine/threonine is required for complete activation of these kinases and initiates specific signaling pathways that ultimately lead to many of the cellular responses associated with PI 3-K [32]. Each kinase phosphorylated by PDK-1 therefore controls specific signaling pathways in time and space, placing PDK-1 at the apex of complex networks of intracellular signaling. The purpose of our study was therefore to determine whether PDK-1 plays a role in an *in vitro H. pylori* infection model with a human gastric epithelium cell line.

## Methods

### Cell culture

The human gastric adenocarcinoma cell line, AGS (ATCC CRL 1739) was grown in RPMI 1640 medium (Cellgro, Herndon, VA) supplemented with 10 % heat inactivated fetal calf serum (FCS, HyClone Laboratories, Logan, UT) and 100 U/ml penicillin/ 100 μg/ml streptomycin (Pen/Strep; Cellgro) and incubated at 37 °C with 5 % CO_2_. Twenty four hours prior to infection with *H. pylori*, cells were washed and cultured in antibiotic-free medium at a concentration of 5 × 10^5^ cells/ml. AGS cells were infected with various strains of *H. pylori* (SS1, 26695, 60190, G27, and SD4; each at a multiplicity of infection, MOI of 100) for 24 h. Additionally, AGS cells were infected with heat-killed *H. pylori* (60190) at a concentration equivalent to an MOI of 100. Cells were also treated with the Src inhibitor, PP2 (10 μM) for 24 h.

### *H. pylori* strains

*H. pylori* strains used in this study including their virulence traits are listed in Table 1. *H. pylori* were routinely maintained on solid medium, Columbia agar (Becton Dickinson, MD) supplemented with 5 % laked blood and grown at 37 °C under microaerophilic conditions (5 % O_2_, 10 % CO_2_, 85 % N_2_) as previously described [33]. Bacteria used to infect gastric epithelial cells were subcultured into liquid medium, brain heart infusion broth (BHI, Becton Dickinson) supplemented with 5 % FCS and cultured for 24 h on a reciprocal shaker at 37 °C under microaerophilic conditions. Before infections, spiral bacteria were enumerated using a Petroff-Hausser chamber and added to gastric cells at an MOI of 100. Bacteria used for infections were in the logarithmic phase of growth. To heat-inactivate *H. pylori*, bacteria were heated at 100 °C for 10 min.

**Table 1:** *H. pylori* strains used in this study

Name	Virulence traits	Reference
60190	*vacA* (s1a/m1) and *cagA* positive	[47]
26695	*vacA* (s1b/m1) and *cagA* positive	[63, 64]^a^
G27	*vacA* (s1b/m1) and *cagA* positive	[65, 66]^a^
SS1	*vacA* (s2/m2) and *cagA* positive	[63, 67]
SD4	*vacA* and *cagA* positive	[68]

^a^Strain has been sequenced

### SDS-PAGE and Western blotting

Both attached and detached gastric epithelial cells were harvested, washed in phosphate buffered saline (PBS) three times, lysed in buffer (20 mM HEPES, pH 7.5, 2 mM EDTA, 2 mM EGTA, 5 mM MgCl_2_, 300 μM phenylmethylsulfonyl fluoride, 1 mM vanadate, 40 μg/ml leupeptin, and 1 μM microcystin), sonicated, and resuspended in Laemmli sample buffer. The proteins were separated by sodium dodecyl sulfate-polyacrylamide gel electrophoresis (SDS-PAGE) in 10 % gels and transferred to polyvinylidinedifluoride membranes (PVDF, Bio-Rad, Hercules, CA). Membranes were probed with the antibodies to phospho Ser 241 PDK-1, total PDK-1 protein, phospho Ser 473 Akt, total Akt protein, or heat shock protein 27 (Hsp27) which were all purchased from Cell Signaling Technologies (Danvers, MA). Proteins were detected using chemiluminescence and quantified with a CCD camera using an Alpha InnotechFluorQ bio-imaging system.

### Generation of His-tagged PDK-1

His-tagged PDK-1 WT was expressed and purified from baculovirus-infected Sf21 cells. Sf21 cells were maintained in Hink’s TNM-FH medium (Cellgro), supplemented with 10 % FCS and 1 % penicillin/streptomycin, and incubated for 4 days with a baculovirus encoding His-PDK-1. Purification was conducted using the IMAC purification kit on Profinia (Bio-Rad), and purity was assessed by Coomassie staining of SDS gels. Whole *H. pylori* lysates were directly incubated with purified recombinant His_6_-PDK-1 for up to 60 min.

### Statistical analysis

Data are represented as mean ± standard deviation. Data from different groups were compared statistically using two-tailed Student’s *t* test. *P* values of less than 0.05 were considered statistically significant.

## Results

### Effect of *H. pylori* infection on PDK-1-activity in gastric epithelial cells

**Fig. 1:**
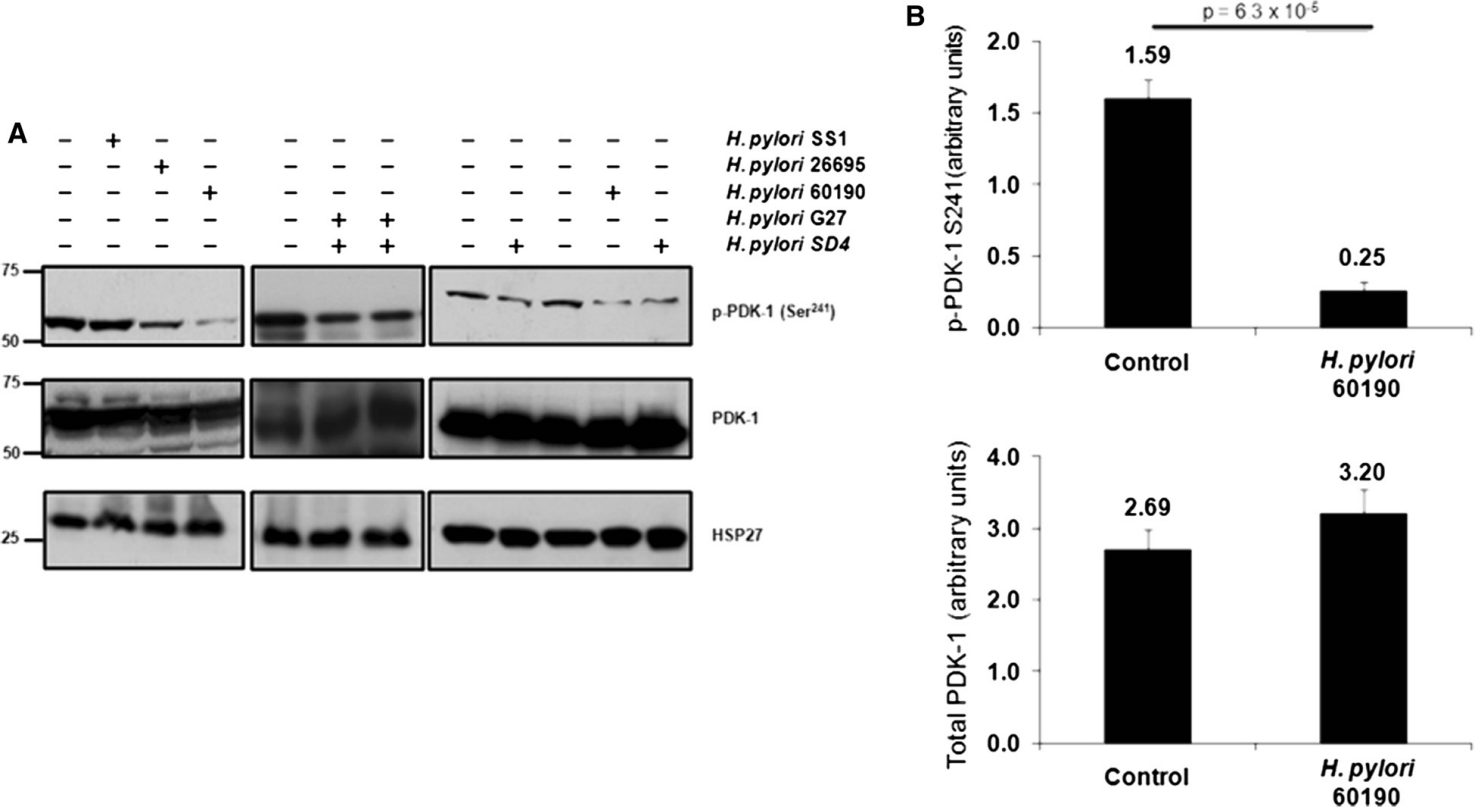
Incubation of *H. pylori* with AGS cells resulted in dephosphorylation of PDK-1 at Ser 241. **a** After infection with *H. pylori* strains SS1, 26695, 60190, G27, or SD4, total AGS protein was separated by SDS-PAGE followed by Western blot to detect phopho Ser 241 PDK-1 (upper panel) and total PDK-1 protein (lower panel). **b** *H. pylori*, strain 60190 incubated with AGS cells for 24 h and PDK-1 phosphorylation at Ser 241 and total protein levels were quantified. Both phospho Ser 241 and total protein levels were normalized to the housekeeping protein Hsp27. The amount of phosphorylation or total PDK-1 protein is expressed as a ratio of control protein, i.e. (phospho protein/Hsp27)/(total protein/Hsp27). Data are representative of at least 5 different experiments and presented as a percent of control and indicate the relative amount of phosphorylation or protein remaining in the system

Secretion of CagA by *H. pylori* into human cells through the T4SS, has previously been demonstrated to hijack intracellular signaling systems involving the tyrosine kinase Src, the tyrosine phosphatase SHP-2, and the adaptor protein Grb2 [34, 35]. We identified Src and Grb2 as PDK-1binding proteins through a proteomic screen (King, unpublished results), suggesting that PDK-1 activity may also be regulated in response to *H. pylori* infection. In initial studies, we wanted to determine whether incubation of *H. pylori* with a gastric epithelial cell line, AGS could directly modulate signaling through PI 3-kinase. Cells were infected for 24 h with five different *H. pylori* strains: SS1, 26695, 60190, G27, and SD4. Incubation of cells with *H. pylori* resulted in dephosphorylation of endogenous PDK-1 at Ser 241 (Fig. 1a, upper panel). The extent of dephosphorylation of PDK-1 was dependent upon the strain of *H. pylori* used, with the 60190 strain being the most effective. It was possible that incubation of *H. pylori* with AGS cells simply activated destruction of the PDK-1 protein, which would explain the loss of phosphorylation at serine 241. To test this, we stripped the blot and re-probed for total PDK-1 protein (Fig. 1a, lower panel). Although we did see some proteolytic clipping of the PDK-1 protein, it largely remained intact, suggesting that *H. pylori* infection of AGS cells resulted in dephosphorylation of PDK-1. Next, we measured the activity of endogenous PDK-1 in AGS cells after incubation with the most effective *H. pylori* strain, 60190 for 24 h. There was an 85 % decrease in Ser 241 phosphorylation but no significant loss of PDK-1 protein (Fig. 1b).

### Infection of AGS cells with *H. pylori* results in altered phosphorylation of Akt

**Fig. 2:**
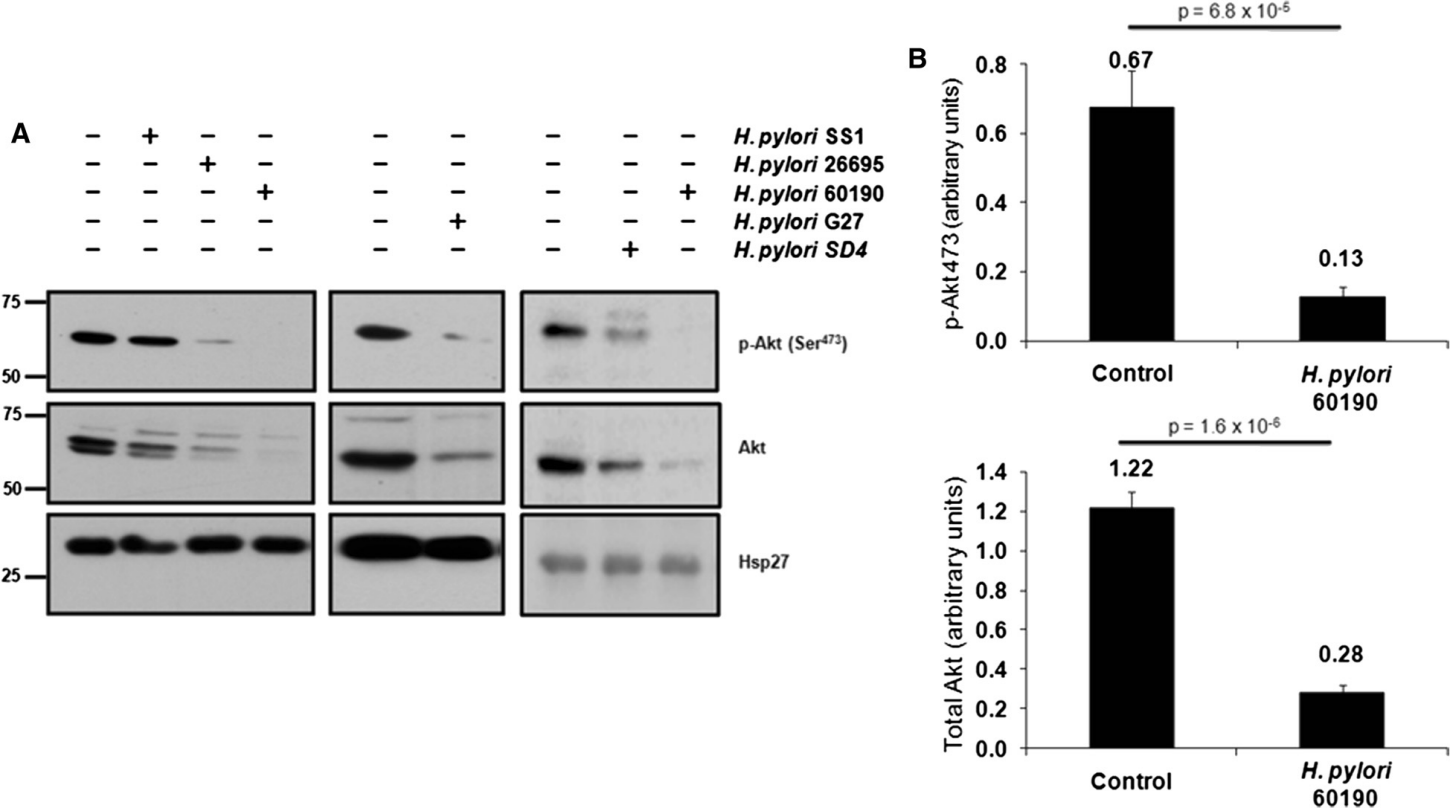
*H. pylori* associated dephosphorylation of PDK-1 alters the phosphorylation stability of Akt. **a** After infection with *H. pylori* strains SS1, 26695, 60190, G27, or SD4, total AGS protein was separated by SDS-PAGE followed by Western blot to detect phopho Ser 473 Akt (upper panel), total Akt (middle panel), or Hsp27 (bottom panel). **b** *H. pylori*, strain 60190 was incubated with AGS cells for 24 h and Akt phosphorylation at Ser 473 and total protein levels were quantified. Both phospho Ser 473 and total protein levels were normalized to the housekeeping protein Hsp27 and expressed as a ratio of control protein, i.e. (phospho protein/Hsp27)/(total protein/Hsp27). Data are representative of at least 3 different experiments and presented as a percent of control and indicate the relative amount of phosphorylation or protein remaining in the system

Phosphorylation of PDK-1 at Ser 241 has been reported to be constitutive and necessary for phosphorylation of downstream substrates [36]. Therefore, we wanted to determine whether incubation of AGS with the *H. pylori* strain 60190, which robustly desphosphorylated Ser 241 had an effect on Akt. Endogenous Akt activity was measured in AGS cells after incubation with *H. pylori* 60190 for 24 h. Incubation of cells with *H. pylori* resulted incomplete dephosphorylation of endogenous Akt at Ser 473 (Fig. 2a, upper panel). Interestingly, the Akt protein was substantially and specifically degraded upon treatment with *H. pylori* (Fig. 2a, middle panel), but a control protein, Hsp27 was not affected. An 80 % decrease in Ser 473 phosphorylation and 78 % decrease in Akt protein were observed in cells incubated with *H. pylori* 60190 (Fig. 2b).

### Mechanism of *H. pylori* dephosphorylation of PDK-1

**Fig. 3:**
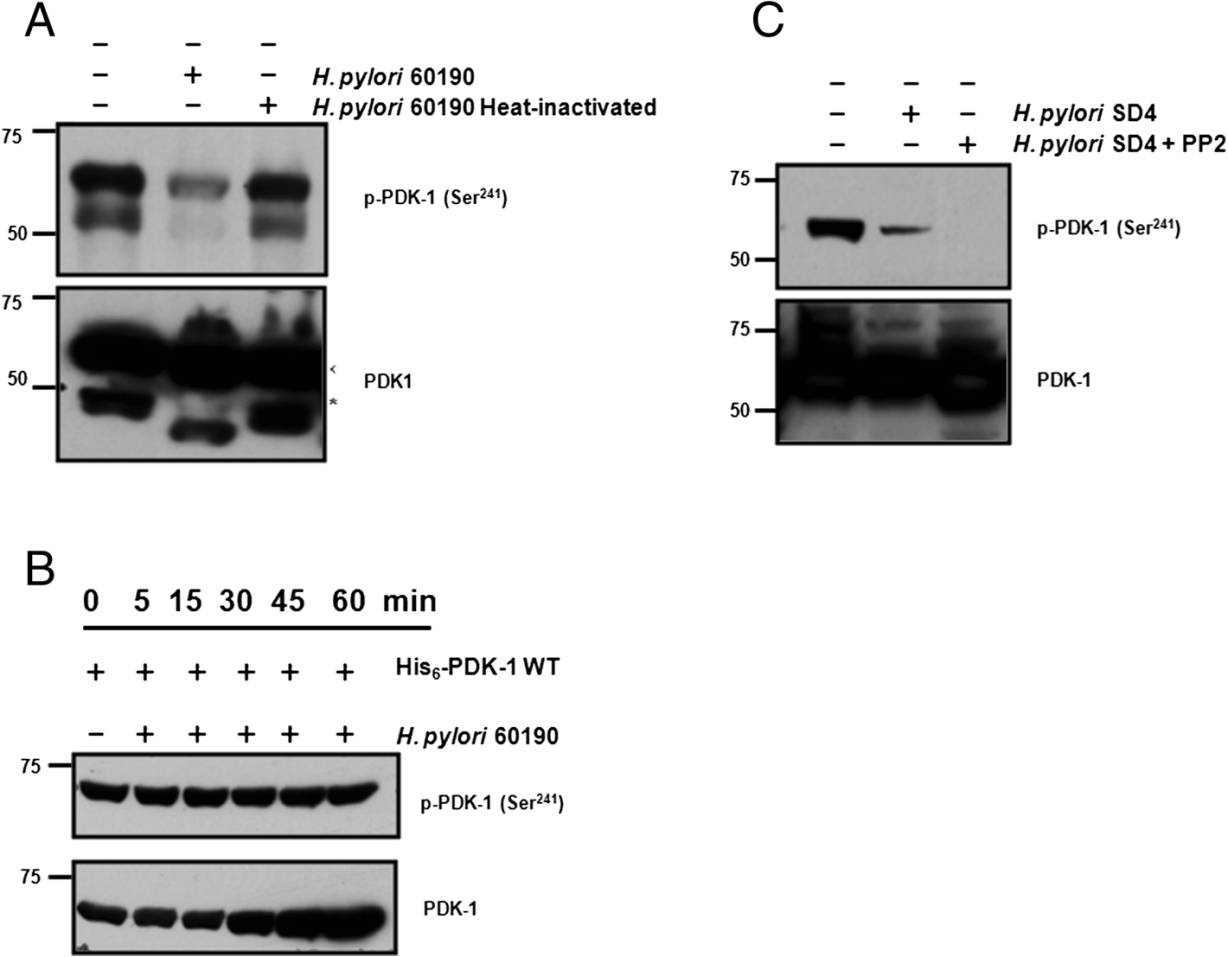
PDK-1 dephosphorylation in the presence of *H. pylori* is indirect. **a** Representative Western blot of phospho Ser 241 PDK-1 and total PDK-1 protein following incubation of AGS cells with live (lane 2) or heat-killed *H. pylori* (lane 3). Total PDK-1 protein ran as two distinct species, one at the expected molecular weight (arrow) and at a faster migrating band (*) that was likely proteolytically cleaved. **b** Incubation of whole *H. pylori* lysates with purified recombinant His_6_-PDK-1. **c** Incubation of AGS cells with *H. pylori* in the absence or presence of Src inhibitor, PP2

To further explore the effect of *H. pylori* on PDK-1 activity, *H. pylori* strain 60190 was used. Endogenous PDK-1 Ser 241 was robustly dephosphorylated upon incubation of AGS cells with *H. pylori* 60190, but not in cells incubated with heat-inactivated *H. pylori* 60190, indicating that the *H. pylori* effect on PDK-1 was proteinaceous (Fig. 3a). To determine whether *H. pylori* directly secreted a protein phosphatase that dephosphorylates PDK-1, purified recombinant His_6_-PDK-1 generated in baculovirus and constitutively phosphorylated at Ser 241, was incubated with whole *H. pylori* lysate for up to 1 h (Fig. 3b). At various times, His_6_-PDK-1 was removed from the incubation mixture and phosphorylation at Ser 241 was verified by Western blotting. No significant decrease in phosphorylation over the 1 h time course was observed, suggesting that PDK-1 is not the direct target of a secreted *H. pylori* protein.

To determine whether signal transduction through Src, a previously known *H. pylori* target in mammalian cells, was indirectly responsible for the dephosphorylation of PDK-1, AGS cells were next incubated with *H. pylori*, SD4. We switched to SD4 because this strain previously yielded a strong hummingbird effect (data not shown). Therefore, this made it an efficient system to study the effects of Src. AGS cells were incubated with *H. pylori* SD4 for 24 h in the absence or presence of a Src inhibitor, PP2 (Fig. 3c). As expected control cells incubated without *H. pylori*, had robust Ser 241 phosphorylation, while cells incubated with *H. pylori* had decreased Ser 241 phosphorylation. Treatment with PP2 did not abrogate PDK-1 dephosphorylation. As a control that PP2 was working, the AGS cells did not display the typical ‘hummingbird’ phenotype (data not shown), which is known to be Src dependent [10, 34, 37]. Taken together, these results suggest that the effect of *H. pylori* on PDK-1 Ser 241 phosphorylation is not a Src-mediated event.

### The role of *H. pylori* type IV secretion system in PDK-1dephosphorylation

**Fig. 4:**
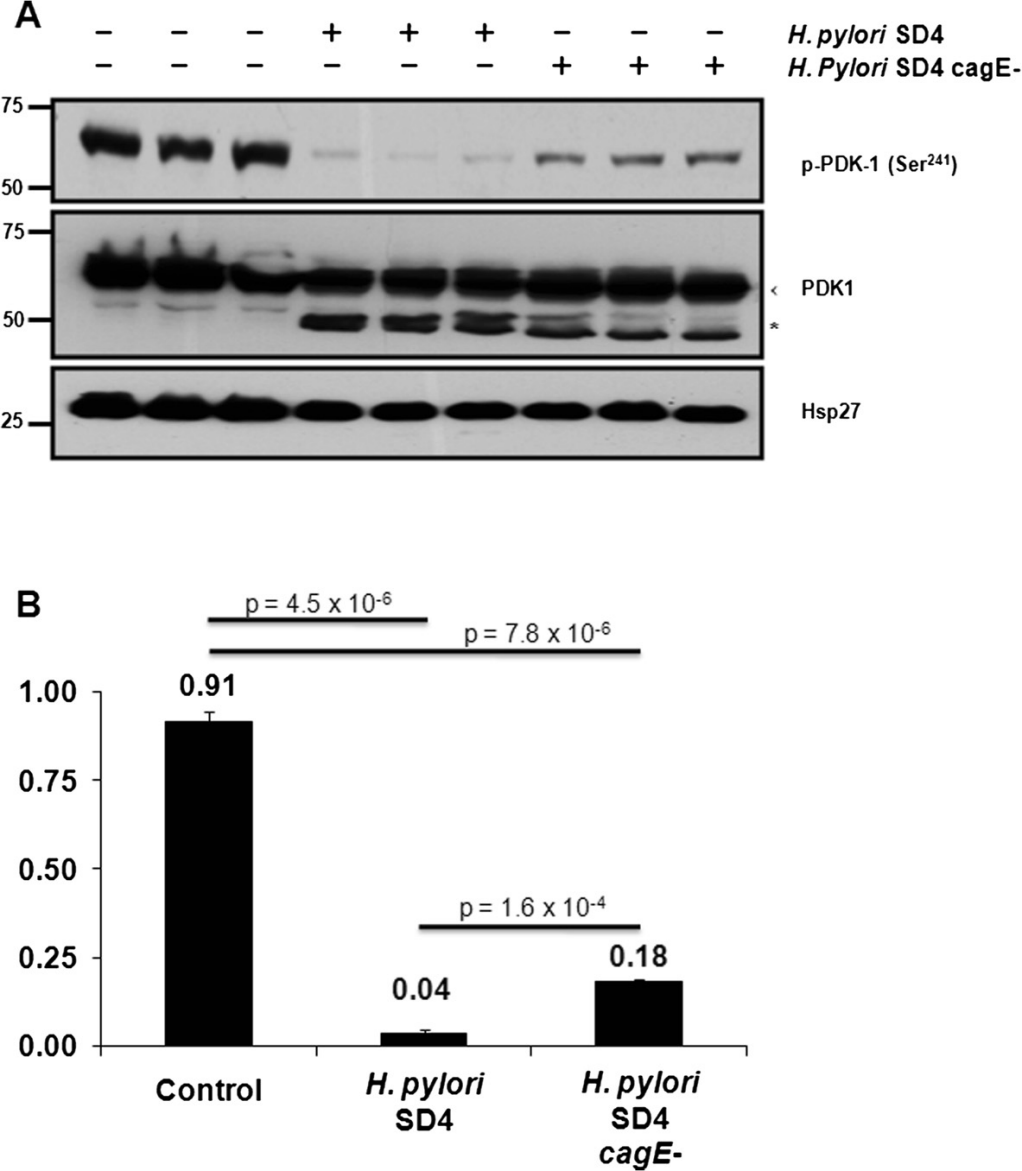
*H. pylori* type IV secretion system is not required for complete PDK-1 dephosphorylation. **a** AGS cell lysates were separated by SDS-PAGE followed by Western blotting with antibodies to phospho Ser 241 PDK-1 (upper panel), total PDK-1 protein (middle panel), or Hsp27 (lower panel). Total PDK-1 protein ran as two distinct species, one at the expected molecular weight (arrow) and at a faster migrating band (*) that was likely proteolytically cleaved. **b** PDK-1 phosphorylation levels and total protein were quantified by normalizing to Hsp27 levels and the amount of phosphorylation or total protein expressed as a ratio of control protein, i.e. (phospho protein/Hsp27)/(total protein/Hsp27). Data are representative of at least 3 different experiments and presented as a percent of control and indicate the relative amount of phosphorylation or protein remaining in the system

We next wanted to determine whether disruption of the T4SS machinery could alter the ability of *H. pylori* to modulate PDK-1 phosphorylation. Minimal PDK-1 dephosphorylation observed in the SS1 strain (Fig. 1a), which is reported to have a non-functional T4SS [38] suggests that a defect in the ability of *H. pylori* to deliver CagA into the target cell might be involved in this effect. To test this, we used CagE deficient *H. pylori* (SD4 *cagE*-), which are unable to deliver CagA protein into gastric epithelial cells [39, 40]. Triplicate samples of AGS cells were infected with either wild type SD4 *H. pylori* strain or SD4 *cagE*- for 24 h and the phosphorylation state of PDK-1 was monitored by Western blot (Fig. 4a top panel). Almost complete dephosphorylation (96 %) of endogenous PDK-1 at Ser 241 was observed in cells infected with the wild type SD4 *H. pylori* (Fig. 4b). In cells infected with SD4 *cagE*-, residual PDK-1 phosphorylation was detected, but was also greatly reduced (>80 %) suggesting that the T4SS plays a minor role in this process (Fig. 4a, top panel and Fig. 4b). Reprobing with PDK-1 again showed that the protein remained largely intact, however, partial clipping of PDK-1 protein was observed in the presence of wild-type and *cagE* deficient *H. pylori* (Fig. 4a, middle panel). A Western blot of Hsp27 is shown in the bottom panel as a loading control (Fig. 4a, bottom panel). Because only partial recovery of PDK-1 phosphorylation was observed in the presence of the *cagE*- mutant suggests that dephosphorylation of PDK-1 is not entirely dependent on the *H. pylori* T4SS.

## Discussion

Disruption of cell survival signaling pathways leads to inappropriate cellular proliferation, growth, and survival, which has been implicated in the genesis and/or progression of numerous human cancers, including melanoma, breast, colon, pancreatic, prostrate, ovarian, lung, and gastric cancers [41–43]. Host signaling pathways play significant roles in the pathogenesis of *H. pylori* disease. We used the gastric adenocarcinoma cell line, AGS in the present study. The use of this cell line is invaluable and has been used as a standard model to study effects of *H. pylori* infection in gastric epithelial cells [44–48]. However, there are limitations of using this cell line given that it is a gastric cancer cell line and as such does not always represent normal cell physiology. Here, we showed that incubation of gastric epithelial cells with *H. pylori* for 24 h resulted in dephosphorylation of PDK-1. This study is the first to describe conditions that result in dephosphorylation of the PDK-1 activation loop in response to a biological stimulus. PDK-1 is pivotal in cellular functions such as proliferation, cell cycle entry, cell survival, and cytoskeletal rearrangements [22, 49].

Molecular events that initiate *H. pylori*-induced gastric cancer are not clearly known. Altered cell signaling mechanisms that affect cell growth and differentiation of the gastric epithelium have been suggested to be the underlying cause of gastric cancer [50]. Our data suggest that an uncharacterized *H. pylori* protein is required for an intracellular mammalian phosphatase to dephosphorylate PDK-1. Evidence for this comes from experiments with heat-killed *H. pylori* that do not alter the phosphorylation state of PDK-1 (Fig. 3a) and from experiments where purified recombinant His_6_-PDK-1 was not dephosphorylated upon incubation with *H. pylori* lysates (Fig. 3b). Further, use of the Src kinase inhibitor PP2 indicated that dephosphorylation of PDK-1 was independent of Src activity (Fig. 3c). Together, these results suggest that a host factor mediates PDK-1 dephosphorylation in response to infection with *H. pylori*.

We also found that *H. pylori* dephosphorylation altered the phosphorylation status and protein stability of the PDK-1 substrate, Akt. Our observation of *H. pylori* dephosphorylation of Akt is not in disagreement with previously published studies that show *H. pylori* activate Akt [51, 52]. This is primarily due to the difference is the time point selected for infection. Indeed, using gastric epithelial cells Nagy et al. [51] demonstrated in a very elegant study that *H. pylori* induction of Akt activation was dependent on time. The highest activation was at 2 h post infection with *H. pylori* and by 24 h, the time used in these studies, induction of Akt activation was very low [51]. All these studies to date, including our present study, report that *H. pylori* infection regulates Akt activity.

Severity and progression of *H. pylori* disease has been linked to *H. pylori* virulence factors [7, 8]. We showed dephosphorylation of PDK-1 occurred in the presence of *H. pylori* lacking the virulence factor, *cagE*. Although there was a statistically significant difference in the amount of phosphate detected at Ser 241 in PDK-1 between cells incubated with wild type SD4 and SD4 *cag*E- mutant (Fig. 4b), a majority (>80 %) of phosphate was removed under both conditions. This suggests that an intact T4SS played only a limited role in this process.

*H. pylori* dephosphorylation of PDK-1 and subsequent dephosphorylation of its substrates, including Akt, which regulates cell survival may therefore result in an imbalance between proliferation and apoptosis. The process of *H. pylori*-related apoptosis in involvement of gastric cancer remains controversial. While there is a consensus that increased cell proliferation favors tumorigenesis, the process that leads to the increased cell proliferation is contradictory. Some studies using animal models have reported induction of apoptosis by *H.* p*ylori* during the early stages of infection followed later by increased cell proliferation, which correlates well with what has been observed in humans infected with *H. pylori* [53, 54]. Overall, implications from our present study agree with the observation that there is an initial induction of apoptosis during the early infection with *H. pylori*, which leads to compensatory heightened cell proliferation associated with the development of gastric cancer. The assumption that reduced PDK-1 phosphorylation is associated with *H. pylori*-induced apoptosis could be proven during the course of *H. pylori* infection. Hence, further investigation is required to better understand how PDK-1 activity changes during *Helicobacter* disease. Nonetheless, our findings clearly demonstrate that *H. pylori* regulates PDK-1 phosphorylation at the activation loop serine, which is key for enzyme function. Although we cannot draw direct conclusions that PDK-1 dephosphorylation plays a role in *H. pylori*-induced gastric cancer, we show that incubation with *H. pylori* resulted in dephosphorylates PDK-1 protein. PDK-1 dephosphorylation is associated with induction of apoptosis [55–58]. Further, apoptosis affects the rate of new cell proliferation thereby disrupting the balance between these cellular events, a process implicated in *H. pylori*-associated carcinogenesis [59–62]. Our data provide information on cellular responses that mediate *H. pylori* infection in gastric epithelial cells.

## Conclusions

Our study provides evidence that *H. pylori* dephosphorylates PDK-1, which alters phosphorylation and stability of the anti-apoptotic PDK-1 substrate, Akt. We show that activation loop phosphorylation of PDK-1, which plays a central role in cell survival signaling pathways, is dysregulated in response to *H. pylori* infection. This dysregulation could modify signaling responses of the host including altered rates of apoptosis and cell proliferation, which may contribute to *H. pylori*-induced gastric carcinogenesis. Imbalance between apoptosis and cell proliferation in gastric mucosal epithelia has been implicated in *H. pylori*-associated gastric carcinogenesis [60–62]. Despite testing for logical *H. pylori* and host proteins, the host protein directly involved in PDK-1 dephosphorylation remains unknown. Our present results allow us to propose that dephosphorylation of host PDK-1 may represent one of the important mechanisms by which *H. pylori* induces the development of gastric cancer.

## Abbreviations

PDK1: 3-phosphoinositide-dependent kinase-1
Akt/PKB: protein kinase B
CagA: Cytotoxin-associated gene A
VacA: Vacuolating cytotoxin A
PAI: Pathogenicity island
TSS4: Type IV secretion system
PI 3-K: Phosphatidylinositol 3-OH-kinase
PI-3,4-P2: Phosphatidylinositol 3,4-bisphosphate
PI-3,4,5-P3: Phosphatidylinositol 3,4,5-trisphosphate
PH: Pleckstrin homology
PKC: protein kinase C
FCS: Fetal calf serum
SS1: Sidney strain 1, MOI, multiplicity of infection
PBS: Phosphate buffered saline, SDS-PAGE, Sodium dodecyl sulfate-polyacrylamide gel electrophoresis
PVDF: Polyvinylidinedifluoride

## References

[1] Covacci A, Telford JL, Del Giudice G, Parsonnet J, Rappuoli R (1999). *Helicobacter pylori* virulence and genetic geography. Science.

[2] Peek RM, Blaser MJ (2002). *Helicobacter pylori* and gastrointestinal tract adenocarcinomas. Nat Rev Cancer.

[3] Kwok T, Zabler D, Urman S, Rohde M, Hartig R, Wessler S, Misselwitz R, Berger J, Sewald N, Konig W (2007). *Helicobacter* exploits integrin for type IV secretion and kinase activation. Nature.

[4] Moss SF, Sood S (2003). *Helicobacter pylori*. Curr Opin Infect Dis.

[5] Murata-Kamiya N, Kurashima Y, Teishikata Y, Yamahashi Y, Saito Y, Higashi H, Aburatani H, Akiyama T, Peek RM, Azuma T (2007). *Helicobacter pylori* CagA interacts with E-cadherin and deregulates the beta-catenin signal that promotes intestinal transdifferentiation in gastric epithelial cells. Oncogene.

[6] Tammer I, Brandt S, Hartig R, Konig W, Backert S (2007). Activation of Abl by *Helicobacter pylori*: a novel kinase for CagA and crucial mediator of host cell scattering. Gastroenterology.

[7] Parsonnet J, Friedman GD, Orentreich N, Vogelman H (1997). Risk for gastric cancer in people with CagA positive or CagA negative *Helicobacter pylori* infection. Gut.

[8] Blaser MJ, Perez-Perez GI, Kleanthous H, Cover TL, Peek RM, Chyou PH, Stemmermann GN, Nomura A (1995). Infection with *Helicobacter pylori* strains possessing cagA is associated with an increased risk of developing adenocarcinoma of the stomach. Cancer Res.

[9] Stein M, Bagnoli F, Halenbeck R, Rappuoli R, Fantl WJ, Covacci A (2002). c-Src/Lyn kinases activate *Helicobacter pylori* CagA through tyrosine phosphorylation of the EPIYA motifs. Mol Microbiol.

[10] Selbach M, Moese S, Hauck CR, Meyer TF, Backert S (2002). Src is the kinase of the *Helicobacter pylori* CagA protein in vitro and in vivo. J Biol Chem.

[11] Bessede E, Dubus P, Megraud F, Varon C (2015). *Helicobacter pylori* infection and stem cells at the origin of gastric cancer. Oncogene.

[12] Bessede E, Staedel C, Acuna Amador LA, Nguyen PH, Chambonnier L, Hatakeyama M, Belleannee G, Megraud F, Varon C (2014). *Helicobacter pylori* generates cells with cancer stem cell properties via epithelial-mesenchymal transition-like changes. Oncogene.

[13] Perez-Perez GI, Salomaa A, Kosunen TU, Daverman B, Rautelin H, Aromaa A, Knekt P, Blaser MJ (2002). Evidence that cagA(+) *Helicobacter pylori* strains are disappearing more rapidly than cagA(−) strains. Gut.

[14] Tsutsumi R, Higashi H, Higuchi M, Okada M, Hatakeyama M (2003). Attenuation of *Helicobacter pylori* CagA x SHP-2 signaling by interaction between CagA and C-terminal Src kinase. J Biol Chem.

[15] Bourzac KM, Guillemin K (2005). *Helicobacter pylori*-host cell interactions mediated by type IV secretion. Cell Microbiol.

[16] Chang YJ, Wu MS, Lin JT, Pestell RG, Blaser MJ, Chen CC. Mechanisms for *Helicobacter pylori* CagA-induced cyclin D1 expression that affect cell cycle. Cell Microbiol. 2006. 10.1111/j.1462-5822.2006.00743.x 16759223

[17] Shibayama K, Doi Y, Shibata N, Yagi T, Nada T, Iinuma Y, Arakawa Y (2001). Apoptotic signaling pathway activated by *Helicobacter pylori* infection and increase of apoptosis-inducing activity under serum-starved conditions. Infect Immun.

[18] Shirin H, Sordillo EM, Oh SH, Yamamoto H, Delohery T, Weinstein IB, Moss SF (1999). *Helicobacter pylori* inhibits the G1 to S transition in AGS gastric epithelial cells. Cancer Res.

[19] Wagner S, Beil W, Westermann J, Logan RP, Bock CT, Trautwein C, Bleck JS, Manns MP (1997). Regulation of gastric epithelial cell growth by *Helicobacter pylori*: offdence for a major role of apoptosis. Gastroenterology.

[20] Hatakeyama M (2004). Oncogenic mechanisms of the *Helicobacter pylori* CagA protein. Nat Rev Cancer.

[21] Martelli AM, Tabellini G, Bressanin D, Ognibene A, Goto K, Cocco L, Evangelisti C (2012). The emerging multiple roles of nuclear Akt. Biochim Biophys Acta.

[22] Toker A, Newton AC. Cellular signaling: pivoting around PDK-1. Cell. 2000;103(2):185–8. doi:S0092-8674(00)00110-0. 10.1016/s0092-8674(00)00110-0 11057891

[23] Casamayor A, Morrice NA, Alessi DR (1999). Phosphorylation of Ser-241 is essential for the activity of 3-phosphoinositide-dependent protein kinase-1: identification of five sites of phosphorylation in vivo. Biochem J.

[24] Cheng X, Ma Y, Moore M, Hemmings BA, Taylor SS (1998). Phosphorylation and activation of cAMP-dependent protein kinase by phosphoinositide-dependent protein kinase. Proc Natl Acad Sci U S A.

[25] Dutil EM, Toker A, Newton AC. Regulation of conventional protein kinase C isozymes by phosphoinositide-dependent kinase 1 (PDK-1). Curr Biol. 1998;8(25):1366–75. doi:S0960-9822(98)00017-7. 10.1016/s0960-9822(98)00017-7 9889098

[26] Kobayashi T, Cohen P (1999). Activation of serum- and glucocorticoid-regulated protein kinase by agonists that activate phosphatidylinositide 3-kinase is mediated by 3-phosphoinositide-dependent protein kinase-1 (PDK1) and PDK2. Biochem J.

[27] Le Good JA, Ziegler WH, Parekh DB, Alessi DR, Cohen P, Parker PJ (1998). Protein kinase C isotypes controlled by phosphoinositide 3-kinase through the protein kinase PDK1. Science.

[28] Pullen N, Dennis PB, Andjelkovic M, Dufner A, Kozma SC, Hemmings BA, Thomas G (1998). Phosphorylation and activation of p70s6k by PDK1. Science.

[29] Alessi DR, James SR, Downes CP, Holmes AB, Gaffney PR, Reese CB, Cohen P (1997). Characterization of a 3-phosphoinositide-dependent protein kinase which phosphorylates and activates protein kinase Balpha. Curr Biol.

[30] King CC, Gardiner EM, Zenke FT, Bohl BP, Newton AC, Hemmings BA, Bokoch GM (2000). p21-activated kinase (PAK1) is phosphorylated and activated by 3-phosphoinositide-dependent kinase-1 (PDK1). J Biol Chem.

[31] Chou MM, Hou W, Johnson J, Graham LK, Lee MH, Chen CS, Newton AC, Schaffhausen BS, Toker A (1998). Regulation of protein kinase C zeta by PI 3-kinase and PDK-1. Curr Biol.

[32] Toker A, Newton AC (2000). Akt/protein kinase B is regulated by autophosphorylation at the hypothetical PDK-2 site. J Biol Chem.

[33] Obonyo M, Sabet M, Cole SP, Ebmeyer J, Uematsu S, Akira S, Guiney DG (2007). Deficiencies of myeloid differentiation factor 88, Toll-like receptor 2 (TLR2), or TLR4 produce specific defects in macrophage cytokine secretion induced by *Helicobacter pylori*. Infect Immun.

[34] Higashi H, Nakaya A, Tsutsumi R, Yokoyama K, Fujii Y, Ishikawa S, Higuchi M, Takahashi A, Kurashima Y, Teishikata Y (2004). *Helicobacter pylori* CagA induces Ras-independent morphogenetic response through SHP-2 recruitment and activation. J Biol Chem.

[35] Mimuro H, Suzuki T, Tanaka J, Asahi M, Haas R, Sasakawa C (2002). Grb2 is a key mediator of helicobacter pylori CagA protein activities. Mol Cell.

[36] Komander D, Kular G, Deak M, Alessi DR, van Aalten DM (2005). Role of T-loop phosphorylation in PDK1 activation, stability, and substrate binding. J Biol Chem.

[37] Bourzac KM, Botham CM, Guillemin K (2007). *Helicobacter pylori* CagA induces AGS cell elongation through a cell retraction defect that is independent of Cdc42, Rac1, and Arp2/3. Infect Immun.

[38] Eaton KA, Kersulyte D, Mefford M, Danon SJ, Krakowka S, Berg DE (2001). Role of *Helicobacter pylori* cag region genes in colonization and gastritis in two animal models. Infect Immun.

[39] Fischer W, Puls J, Buhrdorf R, Gebert B, Odenbreit S, Haas R (2001). Systematic mutagenesis of the *Helicobacter pylori* cag pathogenicity island: essential genes for CagA translocation in host cells and induction of interleukin-8. Mol Microbiol.

[40] Hase K, Murakami M, Iimura M, Cole SP, Horibe Y, Ohtake T, Obonyo M, Gallo RL, Eckmann L, Kagnoff MF (2003). Expression of LL-37 by human gastric epithelial cells as a potential host defense mechanism against *Helicobacter pylori*. Gastroenterology.

[41] Shiojima I, Walsh K (2002). Role of Akt signaling in vascular homeostasis and angiogenesis. Circ Res.

[42] Roy HK, Olusola BF, Clemens DL, Karolski WJ, Ratashak A, Lynch HT, Smyrk TC (2002). AKT proto-oncogene overexpression is an early event during sporadic colon carcinogenesis. Carcinogenesis.

[43] Itoh N, Semba S, Ito M, Takeda H, Kawata S, Yamakawa M (2002). Phosphorylation of Akt/PKB is required for suppression of cancer cell apoptosis and tumor progression in human colorectal carcinoma. Cancer.

[44] Bach S, Makristathis A, Rotter M, Hirschl AM (2002). Gene expression profiling in AGS cells stimulated with *Helicobacter pylori* isogenic strains (cagA positive or cagA negative). Infect Immun.

[45] Cox JM, Clayton CL, Tomita T, Wallace DM, Robinson PA, Crabtree JE (2001). cDNA array analysis of cag pathogenicity island-associated *Helicobacter pylori* epithelial cell response genes. Infect Immun.

[46] Lim JW, Kim H, Kim JM, Kim JS, Jung HC, Kim KH (2004). Cellular stress-related protein expression in *Helicobacter pylori*-infected gastric epithelial AGS cells. Int J Biochem Cell Biol.

[47] Peek RM, Blaser MJ, Mays DJ, Forsyth MH, Cover TL, Song SY, Krishna U, Pietenpol JA (1999). *Helicobacter pylori* strain-specific genotypes and modulation of the gastric epithelial cell cycle. Cancer Res.

[48] Sepulveda AR, Tao H, Carloni E, Sepulveda J, Graham DY, Peterson LE (2002). Screening of gene expression profiles in gastric epithelial cells induced by *Helicobacter pylori* using microarray analysis. Aliment Pharmacol Ther.

[49] Storz P, Toker A (2002). 3′-phosphoinositide-dependent kinase-1 (PDK-1) in PI 3-kinase signaling. Front Biosci.

[50] Houghton J, Stoicov C, Nomura S, Rogers AB, Carlson J, Li H, Cai X, Fox JG, Goldenring JR, Wang TC (2004). Gastric cancer originating from bone marrow-derived cells. Science.

[51] Nagy TA, Frey MR, Yan F, Israel DA, Polk DB, Peek RM (2009). *Helicobacter pylori* regulates cellular migration and apoptosis by activation of phosphatidylinositol 3-kinase signaling. J Infect Dis.

[52] Yan F, Cao H, Chaturvedi R, Krishna U, Hobbs SS, Dempsey PJ, Peek RM, Cover TL, Washington MK, Wilson KT (2009). Epidermal growth factor receptor activation protects gastric epithelial cells from *Helicobacter pylori*-induced apoptosis. Gastroenterology.

[53] Jang TJ, Kim JR (2000). Proliferation and apoptosis in gastric antral epithelial cells of patients infected with Helicobacter pylori. J Gastroenterol.

[54] Peek RM, Wirth HP, Moss SF, Yang M, Abdalla AM, Tham KT, Zhang T, Tang LH, Modlin IM, Blaser MJ (2000). *Helicobacter pylori* alters gastric epithelial cell cycle events and gastrin secretion in Mongolian gerbils. Gastroenterology.

[55] Shehata M, Schnabl S, Demirtas D, Hilgarth M, Hubmann R, Ponath E, Badrnya S, Lehner C, Hoelbl A, Duechler M (2010). Reconstitution of PTEN activity by CK2 inhibitors and interference with the PI3-K/Akt cascade counteract the antiapoptotic effect of human stromal cells in chronic lymphocytic leukemia. Blood.

[56] Shimizu T, Kayamori T, Murayama C, Miyamoto A (2012). Bone morphogenetic protein (BMP)-4 and BMP-7 suppress granulosa cell apoptosis via different pathways: BMP-4 via PI3K/PDK-1/Akt and BMP-7 via PI3K/PDK-1/PKC. Biochem Biophys Res Commun.

[57] Vejux A, Guyot S, Montange T, Riedinger JM, Kahn E, Lizard G (2009). Phospholipidosis and down-regulation of the PI3-K/PDK-1/Akt signalling pathway are vitamin E inhibitable events associated with 7-ketocholesterol-induced apoptosis. J Nutr Biochem.

[58] Perrin AJ, Gunda M, Yu B, Yen K, Ito S, Forster S, Tissenbaum HA, Derry WB (2013). Noncanonical control of C. elegans germline apoptosis by the insulin/IGF-1 and Ras/MAPK signaling pathways. Cell Death Differ.

[59] Xia HH, Talley NJ (2001). Apoptosis in gastric epithelium induced by *Helicobacter pylori* infection: implications in gastric carcinogenesis. Am J Gastroenterol.

[60] Hanahan D, Weinberg RA (2000). The hallmarks of cancer. Cell.

[61] Moss SF (1998). Review article: Cellular markers in the gastric precancerous process. Aliment Pharmacol Ther.

[62] Que FG, Gores GJ (1996). Cell death by apoptosis: basic concepts and disease relevance for the gastroenterologist. Gastroenterology.

[63] Yamaoka Y, Kwon DH, Graham DY (2000). A M(r) 34,000 proinflammatory outer membrane protein (oipA) of *Helicobacter pylori*. Proc Natl Acad Sci U S A.

[64] Tomb JF, White O, Kerlavage AR, Clayton RA, Sutton GG, Fleischmann RD, et al. The complete genome sequence of the gastric pathogen *Helicobacter pylori*. Nature. 1997;388(6642):539–47. doi:10.1038/41483. 10.1038/41483 9252185

[65] de Jonge R, Kusters JG, Timmer MS, Gimmel V, Appelmelk BJ, Bereswill S, et al. The role of *Helicobacter pylori* virulence factors in interleukin production by monocytic cells. FEMS Microbiol Lett. 2001;196(2):235–8. doi:S0378-1097(01)00074-X. 10.1111/j.1574-6968.2001.tb10570.x 11267785

[66] Baltrus DA, Amieva MR, Covacci A, Lowe TM, Merrell DS, Ottemann KM, Stein M, Salama NR, Guillemin K (2009). The complete genome sequence of *Helicobacter pylori* strain G27. J Bacteriol.

[67] Lee A, O’Rourke J, De Ungria MC, Robertson B, Daskalopoulos G, Dixon MF (1997). A standardized mouse model of *Helicobacter pylori* infection: introducing the Sydney strain. Gastroenterology.

[68] Lee A, O’Rourke J, De Ungria MC, Robertson B, Daskalopoulos G, Dixon MF (1997). A standardized mouse model of *Helicobacter pylori* infection: introducing the Sydney strain. Gastroenterology.

